# The Sodium–Glucose Co-Transporter-2 (SGLT2) Inhibitors Reduce Platelet Activation and Thrombus Formation by Lowering NOX2-Related Oxidative Stress: A Pilot Study

**DOI:** 10.3390/antiox11101878

**Published:** 2022-09-22

**Authors:** Pasquale Pignatelli, Francesco Baratta, Raffaella Buzzetti, Alessandra D’Amico, Valentina Castellani, Simona Bartimoccia, Antonio Siena, Luca D’Onofrio, Ernesto Maddaloni, Annachiara Pingitore, Giovanni Alfonso Chiariello, Francesca Santilli, Daniele Pastori, Nicholas Cocomello, Francesco Violi, Maria Del Ben, Vittoria Cammisotto, Roberto Carnevale

**Affiliations:** 1 Department of Clinical, Internal Medicine, Anesthesiological and Cardiovascular Sciences, Sapienza University of Rome, 00185 Rome, Italy; 2 Mediterranea Cardiocentro, 80122 Napoli, Italy; 3 Department of Experimental Medicine, Sapienza University of Rome, 00185 Rome, Italy; 4 Department of Movement, Human and Health Sciences, University of Rome “Foro Italico”, 00135 Rome, Italy; 5 Department of General Surgery and Surgical Specialty Paride Stefanini, Sapienza University of Rome, 00161 Rome, Italy; 6 Cardiovascular Sciences Department, Agostino Gemelli Foundation Polyclinic IRCCS, Catholic University of the Sacred Heart, 00168 Rome, Italy; 7 Department of Medicine and Aging, Center for Advanced Studies and Technology (CAST), “G. d’Annunzio” University Foundation, 66100 Chieti, Italy; 8 Department of Medical-Surgical Sciences and Biotechnologies, Sapienza University of Rome, 40100 Latina, Italy

**Keywords:** gliflozins, type 2 diabetes, oxidative stress, platelet activation, thrombosis

## Abstract

Sodium–glucose co-transporter-2 inhibitors or gliflozins, the newest anti-hyperglycemic class, induce cardioprotective benefits in patients with type 2 diabetes (T2D). As platelet activation and oxidative stress play a key role in atherothrombotic-related complications, we hypothesized that gliflozins might modulate oxidative stress, platelet activation and thrombus formation. We performed an interventional open-label single-arm before-after study in 32 T2D patients on top of their ongoing metformin therapy. The population was divided into two groups: treatment with GLP-1 receptor agonists (GLP-1RA, Group A) and gliflozins (Group B). Oxidative stress, platelet activation and thrombus growth were assessed before and after 15 days of treatment. Compared to the baseline, gliflozins treatment significantly decreased sNOX2-dp (−45.2%, *p* < 0.001), H_2_O_2_ production (−53.4%, *p* < 0.001), TxB2 (−33.1%, *p* < 0.001), sP-selectin (−49.3%, *p* < 0.001) and sCD40L levels (−62.3%, *p* < 0.001) as well as thrombus formation (−32%, *p* < 0.001), whereas it potentiated anti-oxidant power (HBA, +30.8%, *p* < 0.001). Moreover, a significant difference in oxidative stress, platelet activation and thrombus formation across groups A and B was found. In addition, an in vitro study on stimulated platelets treated with gliflozins (10–30 μM) showed a reduction in oxidative stress, platelet activation and thrombus growth. Our results showed that gliflozins have antiplatelet and antithrombic activity related to an NOX2 down-regulation, suggesting a new mechanism responsible for cardiovascular protection.

## 1. Introduction

Type 2 diabetes (T2D) is often complicated by cardiovascular disease (CVD), as shown by the high incidence of acute thrombotic events, such as acute myocardial infarction (MI) and ischemic stroke in these patients [1,2]. Platelet dysfunction plays a central role in the onset of CVD in T2D patients, as these patients disclose an increased thrombotic state related to endothelial dysfunction, coagulation cascade activation and increased platelet reactivity [3,4], contributing to an accelerated atherothrombosis [5]. Moreover, the platelets from T2D patients show high oxidative stress levels due to an increased production of reactive oxygen species (ROS), partly mediated by Nicotinamide adenine dinucleotide phosphate oxidase (NADPH) oxidase 2 (NOX2) activation and, in turn, leading to amplified aggregation [6,7]. In addition, NOX2 is the major source of ROS in cardiomyocytes, and its activation contributes to the ischemia-reperfusion injury that is central to the pathology of major cardiovascular diseases, such as stroke and MI [8]. This evidence accounts for the continuous efforts to improve the antiplatelet strategy in T2D patients with CVD, especially in those undergoing percutaneous coronary interventions (PCI) [9].

Recently, several randomized trials highlighted the cardiovascular benefits associated with new hypoglycemic agents [10,11]. Among the hypoglycemic agents, GLP-1 receptor agonists (GLP-1RA) seem to have a role in reducing platelet aggregation and thrombus formation [12]. In vitro studies demonstrated that GLP-1 metabolites and analogues have an antiplatelet effect thanks to their ability to decrease oxidative stress by improving intracellular anti-oxidant defenses and decreasing ROS production through GLP-1 receptor-dependent and independent pathways [13,14].

The sodium-glucose co-transporter-2 inhibitors (SGLT2i) known as gliflozins (canagliflozin, dapagliflozin and empagliflozin) are the newest drugs approved for the treatment of T2D [15,16].

Beyond the hypoglycemic effect, with a range of glycated hemoglobin reduction of 0.84–1.03% [17,18,19], gliflozins substantially reduce CVD. In clinical trials, canagliflozin [20] and empagliflozin [21] reduced CVD by 14%, empagliflozin reduced CV mortality by 38% [21] and dapagliflozin reduced hospitalization for heart failure and CV mortality by 17% [22].

The mechanisms through which gliflozins produce beneficial effects on the cardiovascular system are partly unclear, and the impact of these new glucose-lowering agents on platelet function has not been completely clarified yet. For example, Lescano et al. demonstrated that the SGLT2 inhibitors synergized with nitric oxide and prostacyclin to reduce human platelet activation [23]. Moreover, chronic treatment with dapagliflozin (25 mg/kg/day, 4 weeks) normalized the glucose and IL-6 levels produced by Kupffer cells and the number of reticulated platelets and megakaryocytes [24].

For this purpose, we hypothesized that gliflozins could have a direct effect on oxidative stress, reducing platelet activation and thrombus formation. By conducting “in vivo” and “in vitro” studies, we evaluated the direct role of SGLT2i and GLP1-RA in platelet function, thrombus formation and NOX2-induced oxidative stress.

## 2. Materials and Methods

### 2.1. Human Study

We performed an interventional single-arm open-label before-after study in T2D patients to investigate the short-term effect (15 days) of gliflozins (dapagliflozin or empagliflozin) or GLP-1RA (semaglutide or dulaglutide) on platelet function, by evaluating Thromboxane B2 (TxB2), soluble P-selectin (sP-selectin) and soluble CD40 ligand (sCD40L); oxidative stress, by evaluating the blood levels of soluble NOX2-derivative peptide (sNOX2-dp), a marker of NOX2 activation and hydrogen peroxide (H_2_O_2_) production; and finally, thrombus formation, by evaluating the total thrombus-formation analysis system (T-TAS).

T2D was diagnosed according to the American Diabetes Association definition [25].

Thirty-two T2D patients were included in the study. The population was divided into two groups: those treated with GLP-1RA (Group A) and those with gliflozins (Group B). The groups were balanced for age, sex, BMI, antiplatelet therapy, statin therapy and smoking habits. GLP-1RA or gliflozins were prescribed according to the clinical indications.

Platelet function, oxidative stress and thrombus formation were assessed at baseline and after 15 days of treatment. Both GLP-1RA and gliflozins were studied as an add-on therapy to metformin.

The patients using any other anti-diabetes treatment were excluded from the study. Other exclusion criteria were: (1) liver insufficiency, (2) advanced chronic kidney disease (eGFR < 30 mL/min/1.73 m^3^), (3) active cancer, (4) recent history (<3 months) of acute vascular events, (5) treatment with anti-oxidant vitamins, (6) diagnosis of type 1 diabetes mellitus, (7) active infection, (8) cardiac arrhythmia or congestive heart failure, (9) use of nonsteroidal anti-inflammatory drugs and (10) age ≥ 80 years.

Informed written consent was obtained from all subjects: the study conformed to the ethical guidelines of the 1975 Declaration of Helsinki and was approved by the local research ethics committee (Rif.6848).

### 2.2. In Vitro Study

#### 2.2.1. Platelet Preparation

The blood samples added with sodium citrated (3.8%, 1/10 (*v*:*v*) were taken from healthy subjects (HS, *n* = 5; males 3, females 2, age 49.0 ± 5.0 years) for the in vitro study in fasting conditions. Blood was centrifuged for 15 min at 180× *g* at room temperature (RT), and the PRP (2 × 10^5^ platelets/μL) was prepared as previously described [26]. Washed platelets (wPLTs) were isolated from the PRP by the next centrifugation (10 min at 300× *g* at RT) and resuspended in Tyrode’s buffer (137 mM NaCl, 2.7 mM KCl, 1.0 mM MgCl_2_, 1.8 mM CaCl_2_, 20 mM HEPES, 0.35% *w*/*v* BSA and 5.6 mM glucose, pH 7.35; Sigma Aldrich, St. Louis, MO, USA). Prostaglandin E1 (PGE1, 1 µM) was added to prevent platelet activation.

#### 2.2.2. Platelet Aggregation

Platelet aggregation (PA) was induced in the wPLT samples by a subthreshold concentration of collagen (STC, 0.25 μg/mL). The concentration of agonists was defined as the highest concentration that elicited a <20% platelet aggregation of collagen as a primer. The PA was measured for 8 min. Before activation, the samples were pre-incubated (20 min at 37 °C) with Dapa (10, 20 and 30 µM) or Dulaglutide (Dula, 100 nmol/L) in the presence of 10 mM of d-Glucose (d-Glu). To determine the PA in wPLTs, we added immediately before to induce aggregation, CaCl_2_ and fibrinogen (1 mM and 100 μg/mL, respectively; Sigma Aldrich, St. Louis, MO, USA). PA was performed with 2 dual-channel modules and the Chrono Log Model 700 LT aggregometer, using the Born method [27]. Finally, the samples were centrifuged for 3 min at 3000 rpm, and the supernatants and pellets were stored at −80 °C for the analysis of TxB_2_, sP-selectin, sCD40L, sNOX2-dp and H_2_O_2_, as reported below.

#### 2.2.3. Serum and Platelet TxB_2_ Production

Plasma and platelets Thromboxane (Tx) A_2_ was analyzed by its stable metabolite, TxB_2_, in the supernatant by an ELISA commercial kit (Cusabio, Houston, TX, USA), according to the manufacturer’s instructions. The values were expressed as pg/mL × 10^8^ cells and pg/mL, respectively. The intra- and inter-assay coefficients of variation for TxB_2_ were <8 and <10%, respectively.

#### 2.2.4. Plasma and Platelet sP-Selectin Levels

The soluble P-selectin (sP-selectin) levels were measured with a commercial immunoassay (DRG International, Springfield, NJ, USA). The intra- and inter-assay coefficients of variation were 4.3% and 6.1% for sP-selectin. The values were expressed in ng/mL for sP-selectin.

#### 2.2.5. Plasma and Platelets Soluble CD40 Ligand Levels

The CD40 Ligand concentrations were measured by an immunoassay solid-phase ELISA (DRG International, Springfield, NJ, USA), designed to measure human soluble CD40 Ligand (sCD40L). The values were expressed as ng/mL, and the intra-assay and inter-assay coefficients of variation were 3.2% and 4.3%, respectively.

#### 2.2.6. Serum and Platelet sNOX2-dp

NOX2 activity was measured in serum and platelets as sNOX2-dp with a previously reported ELISA method [28]. The values were expressed as pg/mL; the intra- and inter-assay coefficients of variation (CV) were <10%.

#### 2.2.7. Serum and Platelets H_2_O_2_ Production

Hydrogen peroxide (H_2_O_2_) was measured by a colorimetric assay as described previously [29]. The final product was read at 450 nm and expressed as μM. The intra- and inter-assay CVs were both <10%.

#### 2.2.8. Determination of % HBA

The hydrogen peroxide (H_2_O_2_) breakdown activity (HBA) was measured with an HBA assay kit (Aurogene, Rome, Italy, code HPSA-50). The % of HBA was calculated according to the following formula: % Of HBA = [(Ac − As)/Ac] × 100, where Ac is the absorbance of H_2_O_2_ 1.4 mg/mL and As is the absorbance in the presence of the serum sample.

#### 2.2.9. Thrombus Formation

The thrombus growth under flow conditions was measured by a thrombus-formation analysis system (T-TAS^®^01 apparatus, Fujimori Kogyo Co., Ltd., Tokyo, Japan) on PL- chips (26 collagen-coated microcapillaries). Whole blood (400 μL), anticoagulated by BAPA (benzylsulfonyl-D-argininyl-prolyl-4-amidinobenzylamide) from T2BM patients at basal (pre-treatment) and after 15 days of therapy (post-gliflozins treatment), or taken from HS or T2D patients and incubated with or without Dapa (30 µM), were collected. Then, 340 μL of the samples were transferred to the PL-chip and analysed. The growth, intensity and stability of the formation of platelet clots were measured by the time needed to reach the occlusion pressure (occlusion time), and the area under the flow-pressure curve (AUC) parameter, which is an area under the pressure curve from the start of the test to a time of 10 min, were studied [30].

#### 2.2.10. Statistical Analysis

Categorical variables were reported as the counts/percentages and the continuous variables as mean ± standard deviation or as the median (interquartile range) according to normal distribution. The comparisons between groups were carried out by Student’s *t* and Mann–Whitney chi^2^ tests when appropriate. The assessment of the treatment effects was made by performing a two-way ANOVA. Bivariate analysis was performed by Pearson and Spearman correlation tests. A value of *p* < 0.05 was considered statistically significant.

All tests were performed using GraphPad Software-Prism7 (San Diego, CA, USA) and IBM SPSS 25.03.

## 3. Results

Thirty-two patients were enrolled, with sixteen for each drug class. The patients in the GLP1-RA arm were prescribed semaglutide (4 patients) and dulaglutide (12 patients). The patients in the gliflozins arm were prescribed empagliflozin (3 patients) and dapagliflozin (13 patients). The two groups were balanced for age, sex, BMI, T2D duration, antiplatelet therapy, statin therapy, metformin dose and smoking habits. The only significant difference was reported in Hb1Ac (7.7 [7.0–8.6] vs. 6.8 [5.9–7.5], *p* = 0.023). The clinical characteristics of T2D patients are reported in Table 1.

### 3.1. In Vivo Study

#### 3.1.1. Oxidative Stress Evaluation

**Figure 1:**
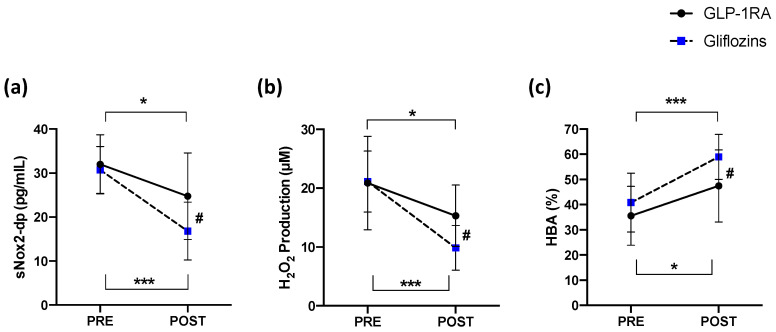
(**a**) Serum soluble NOX2-derived peptide (sNOX2-dp), (**b**) serum H_2_O_2_, and (**c**) blood HBA before and 15 days after administration of GLP-1RA (*n* = 16) or gliflozins (*n* = 16). Data are expressed as mean ± SD. Intra-group significance: * *p* < 0.05; *** *p* < 0.0001; inter-group significance: # *p* < 0.05.

The pairwise comparisons showed that sNOX2-dp and H_2_O_2_ significantly decreased after the treatment of Group A (from 31.97 ± 6.73 to 24.75 ± 9.83 pg/mL, * *p* < 0.05 and from 20.88 ± 7.94 to 15.32 ± 5.21 µM, * *p* < 0.05, respectively) and Group B (from 30.72 ± 5.35 to 16.82 ± 6.56 pg/mL, ** *p* < 0.001 and from 21.14 ± 5.17 to 9.86 ± 3.79 µM, *** *p* < 0.0001, respectively) (Figure 1a,b). Moreover, the pairwise comparisons showed that HBA resulted in significant improvements after treatment for both Group A and Group B (from 35.56 ± 11.68 to 47.44 ± 14.33%, *** *p* < 0.0001 and from 40.81 ± 11.68 to 58.94 ± 8.91%, * *p* < 0.05, respectively) (Figure 1c). A significant difference between the treatments (Group A vs. Group B) was found with respect to sNOX2-dp release (# *p* < 0.05; Figure 1a), H_2_O_2_ production (# *p* < 0.05; Figure 1b) and anti-oxidant activity (HBA) (# *p* < 0.05; Figure 1c). Similar effects for both treatments A and B were found in a subgroup of patients not taking acetylsalicylic acid (ASA), and all were on statin therapy (Figure S1a–c).

#### 3.1.2. Platelet Function

**Figure 2:**
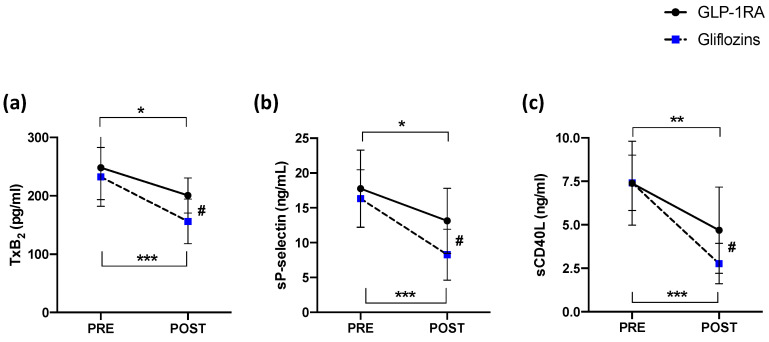
(**a**) Thromboxane B_2_ production, (**b**) soluble P-selectin levels and (**c**) soluble CD40 ligand before and 15 days after administration of GLP-1RA (*n* = 16) or gliflozins (*n* = 16). Data are expressed as mean ± SD. Intra-group significance: * *p* < 0.05; ** *p* < 0.001; *** *p* < 0.0001; inter-group significance: # *p* < 0.05.

Compared to the baseline, platelet TxB2, sP-selectin and the sCD40L levels were significantly reduced in the treatment of Group A (from 248.20 ± 54.75 to 200.50 ± 30.10 pg/mL, * *p* < 0.05; from 17.75 ± 5.54 to 13.14 ± 4.70 ng/mL, * *p* < 0.05; and from 7.40 ± 2.41 to 4.70 ± 2.50 ng/mL, * *p* < 0.05, respectively) and Group B (from 232.31 ± 48.83 to 155.34 ± 37.11 pg/mL, *** *p* < 0.0001; from 16.32 ± 4.14 to 8.27 ± 3.70 ng/mL, *** *p* < 0.0001; and 7.42 ± 1.60 to 2.80 ± 1.20 ng/mL, *** *p* < 0.0001, respectively) (Figure 2a–c). Furthermore, a significant difference between treatments (Group A vs. Group B) was found with respect to TxB2 (# *p* < 0.05; Figure 2a), sP-selectin (# *p* < 0.05; Figure 2b) and the sCD40L levels (# *p* < 0.05; Figure 2c).

Superimposable effects for both treatments A and B were found in a subgroup of patients not taking acetylsalicylic acid (ASA), and all were on statin therapy (Figure S1d–f).

#### 3.1.3. Linear Correlation

The only clinical characteristic correlating with the baseline experimental data was the use of statins, with an inverse correlation with sP-selectin (r = −0.409, *p* = 0.020). The median sP-selectin values in patients using statins or not were 15.7 ± 3.7 vs. 20.1 ± 5.9, *p* = 0.015. None of the baseline characteristics correlates with experimental data ∆ before or after gliflozin administration. Instead, a linear correlation analysis showed that ∆ of sNOX2-dp correlated with ∆ of TxB2 (r = 0.591; *p* = 0.016), ∆ of sP-selectin (r = 0.630; *p* = 0.009) and ∆ of sCD40L (r = 0.511; *p* = 0.043).

#### 3.1.4. Thrombus Formation

**Figure 3:**
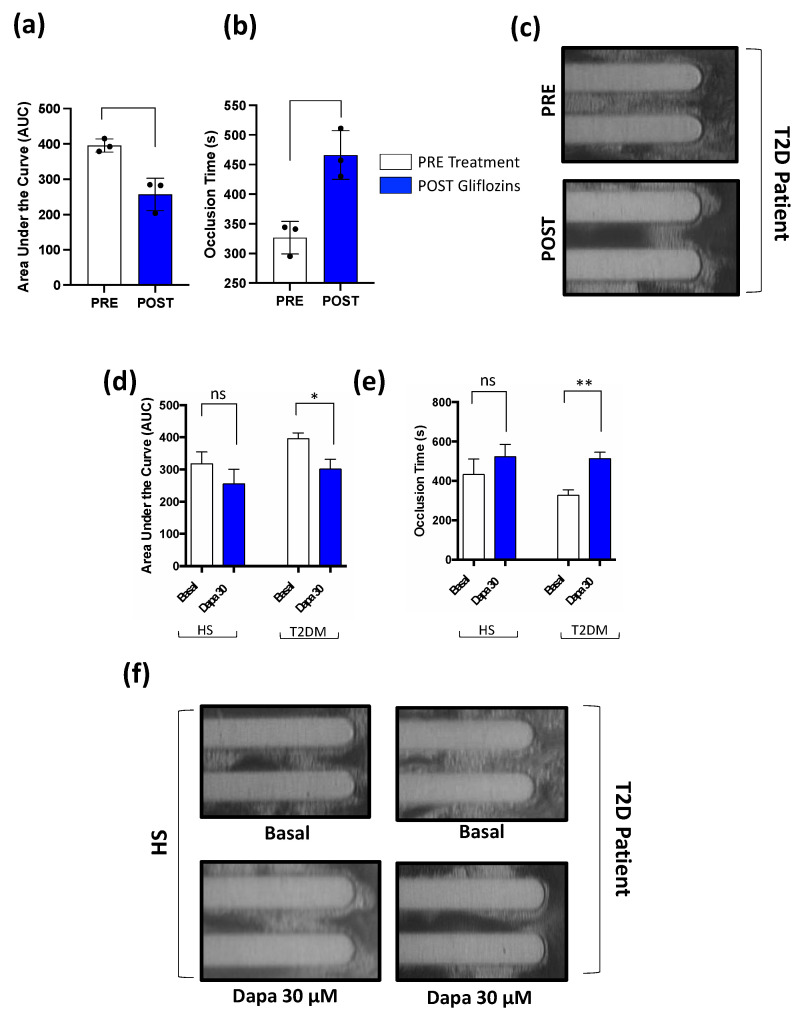
(**a**–**c**) In vivo effect of gliflozins therapy on platelet-dependent thrombus growth under laminar flow. Whole blood samples (T2D, *n* = 3) before treatment (PRE) and after 15 days of gliflozins therapy (POST). (**d**–**f**) In vitro effect of dapagliflozin (Dapa, 30 µM) on platelet-dependent thrombus growth under laminar flow in whole blood samples taken from HS (*n* = 3) and T2D patients (*n* = 3). Samples were analyzed by (**a**,**d**), area under the curve (AUC), and (**b**,**e**) occlusion time; (**c**,**f**), representative images of thrombus formation under laminar flow conditions. Data were expressed as mean ± SD; * *p* < 0.05, ** *p* < 0.01; ns = not significant.

To explore the role of gliflozins in the atherosclerotic process, we evaluated thrombus formation in T2D patients by an experimental model of platelet-dependent thrombus growth under in vivo arterial flow conditions using a microfluidic perfusion assay. This analysis revealed that compared to the baseline, after 15 days of treatment with gliflozins, patients with T2D showed a significant decrease in thrombus growth, as evidenced by a decreased AUC and increased occlusion time (Figure 3a–c). In addition, plasma from T2D patients showed a lower thrombus formation after the in vitro pre-treatment with Dapa (30 µM), as displayed by the increased occlusion time and decreased AUC (Figure 3d–f). These results were not observed in plasma from HS (Figure 3d–f).

### 3.2. In Vitro Study

**Figure 4:**
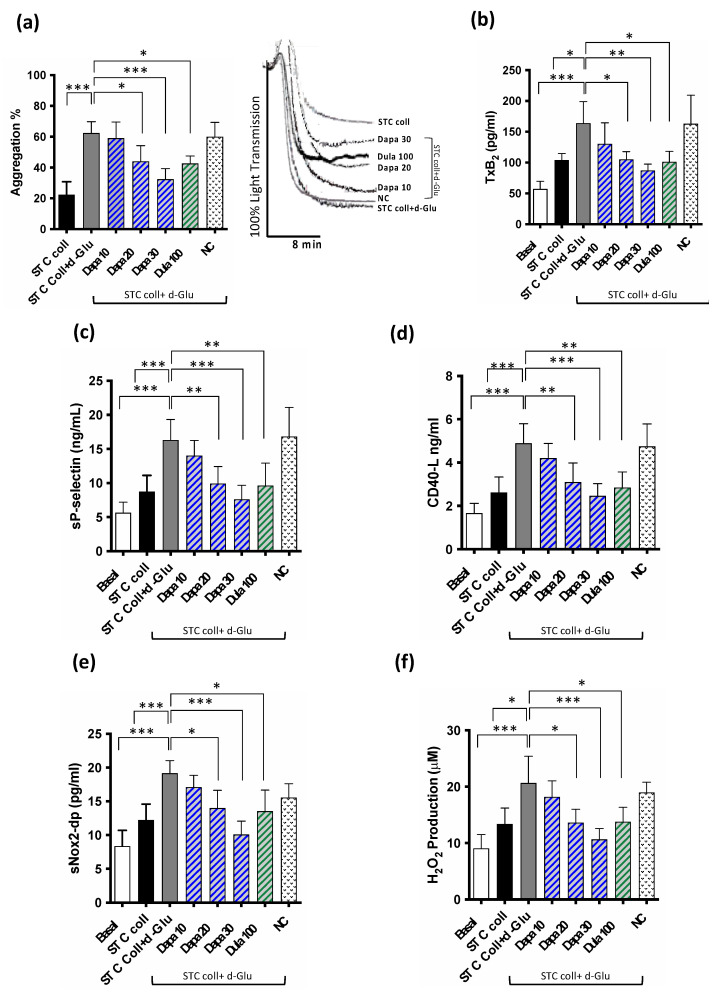
(**a**) Platelet aggregation and representative aggregometer trace, (**b**) Thromboxane B_2_ production, (**c**) soluble P-selectin levels, (**d**) soluble CD 40 ligand, (**e**) serum soluble NOX2-derived peptide (sNOX2-dp), (**f**) serum H_2_O_2_ in washed platelets (wPLTs) taken from HS (*n* = 5) pre-incubated with or without d-Glucose (d-Glu, 10 mM) and then stimulated with or without STC of collagen (0.25 μg/mL) in the presence or less of dapagliflozin (Dapa, 30 µM) or dulaglutide (Dula, 100 nmol/L) or negative control (NC). Data were expressed as mean ± SD; * *p* < 0.05, ** *p* < 0.01; *** *p* < 0.001.

To evaluate the role of gliflozins and GLP-1 agonists on platelet function, oxidative stress and thrombus formation, we performed an in vitro study where the scalar concentration of dapagliflozin (Dapa, 10, 20 and 30 µM) or Dulaglutide (Dula, 100 nmol/L) were incubated with STC collagen-stimulated platelets in the presence of d-Glucose (d-Glu). The incubation of d-Glu with platelets primed with STC collagen resulted in increased platelet aggregation, TxB2 biosynthesis, sP-selectin and CD40L release, along with NOX2 activation and H_2_O_2_ production when compared to the platelets stimulated with STC collagen alone (Figure 4a–f). These effects were significantly inhibited by the pre-treatment with Dapa at 20 µM and 30 µM (Figure 4a–f). Similar results were observed with the pre-treatment with Dula 100 nmol/L. Notably, no change was observed at a concentration of 10 µM of Dapa (Figure 4a–f).

## 4. Discussion

This study provides evidence that gliflozins used in T2D patients significantly improve platelet function and thrombus formation. This effect may be related to a down-regulation of NOX2-mediated oxidative stress (Graphical Abstract). These findings support the hypothesis that gliflozins may provide a cardiovascular benefit, not only through the lowering of blood glucose but also by targeting other pathogenetic pathways involved in the evolution of atherosclerosis.

In our in vitro and in vivo studies, we confirmed and extended the previous evidence [10,11], demonstrating that therapy with GLP-1RA was able to blunt oxidative stress by reducing the NOX2 levels and platelet activation by reducing Thromboxane production and the sP-selectin and sCD40L levels. Overall, the current data in the literature identifies an important role of these drugs in the regulation of platelet activation/aggregation but does not provide direct evidence on the role of arterial thrombosis.

In addition, our sub-analyses, conducted in patients homogeneous for aspirin and statin therapy, confirm the anti-oxidant and antiplatelet effect of both hypoglycemic agent classes after excluding two important confounding factors.

A recent study demonstrated that the SGLT2 inhibitors synergize with nitric oxide and prostacyclin to reduce human platelet activation [23]. In addition, Kohlmorgen et al. showed that dapagliflozin mediated atheroprotection in mice and ameliorated thrombin-platelet-mediated inflammation [31]. Furthermore, Spigoni et al. proved, by an in vitro study, that empagliflozin and dapagliflozin ameliorated lipotoxic damage in stearate-treated myeloid angiogenic cells and reduced ADP-stimulated PLT activation [32]. To date, however, these studies do not completely explain how gliflozins produce a beneficial effect on oxidative stress, platelet function and thrombus formation in T2D. Our study demonstrates, for the first time, a protective mechanism by gliflozins on thrombus formation in T2D. In particular, we observed that after 15 days of intervention with gliflozins (dapagliflozin or empagliflozin), diabetic patients displayed reduced oxidative stress, such as s-NOX2-dp and H_2_O_2_ compared to the patients treated with GLP-1RA. The beneficial effect of these drugs on oxidative status is corroborated by the significant increase in the percentage of HBA, confirming the restoration of a better-balanced redox status. Moreover, dapagliflozin can restore platelet activation, as indicated by the decrease in thromboxane formation and sP-selectin and sCD40L levels.

The study has pathophysiologic and clinical implications. The up-regulation of NOX2 in T2D has important functional consequences, as it is associated with enhanced oxidative stress and ultimately with platelet activation. This finding supports and extends previous data indicating that platelet ROS formation is implicated in the thrombus formation process [33] and provides further insight into the association between T2D and thrombosis.

Putting these findings together with the safety profile of gliflozins when used in non-diabetic patients [34,35], we could speculate a possible use of these drugs in T2D patients having a good metabolic compensation and/or in other non-diabetic populations with cardiovascular disease. However, this hypothesis needs to be confirmed by ad hoc studies.

The present study also has limitations that should be acknowledged. As this study’s population was relatively small and observational in nature, the validity of the conclusions offered needs to be tested in a larger population before any revision of guidelines can be recommended. In addition, the comparison between the two drug classes might be affected by the differences in glycemic compensation between the groups, as patients treated with GLP1-RA showed a higher baseline Hb1Ac. However, our univariate analyses showed no correlation between the baseline Hb1Ac levels and the anti-oxidant and antiplatelets gliflozins effect. Another limitation of the study is the absence of data on metabolic control after 15 days of treatment with gliflozins and GLP1-RA.

Our findings need to be confirmed in a larger population by a randomized study of extended duration (at least 3 months) to assess whether gliflozins may also be considered a valid anti-oxidant and antiplatelet therapy in diabetic patients.

In addition, our results could rise to new studies evaluating the role of SGLT2i in the mechanisms that can favor the atherothrombotic process, such as endothelial function. Previous studies demonstrated a positive effect of SGLT2i on endothelial function [36,37] and increased mitochondrial oxidative stress with the up-regulation of anti-oxidant mitochondrial enzymes in the bone marrow compartment [38]. Thus, future studies could investigate the mechanism by which new hypoglycemic agents modulate the endothelial function and the possible role of these drugs as mitochondrial function modulators.

## 5. Conclusions

Overall, the results from this study suggest that gliflozins decrease platelet activation and thrombus formation by an NOX2 down-regulation mechanism.

## Figures and Tables

**Table 1:** Clinical characteristics of the population.

	GLP1-RA Group A (*n* = 16)	Gliflozins Group B (*n* = 16)	*p*-Value
*Age* (*years*)	59.9 ± 10.2	57.5 ± 5.7	0.432
*Women* (*n*, *%*)	4, 25.0%	3, 18.8%	0.669
*Diabetes duration* (*years*)	4.0 [1.0–7.0]	4.5 [3.0–9.0]	0.426
*BMI* (*kg*/*m*^2^)	29.9 ± 4.2	30.7 ± 4.7	0.597
*Smoking* (*n*, *%*)	4, 25.0%	4, 25.0%	-
*Arterial hypertension* (*n*, *%*)	10, 62.5%	11, 68.8%	0.710
*Statin* (*n*, *%*)	11, 68.8%	11, 68.8%	-
*Acetylsalicylic acid* (*n*, *%*)	4, 25.0%	3, 18.8%	0.669
*Metformin Dose* (*g*)	2.0 ± 0.2	1.8 ± 0.6	0.317
*Glycaemia* (*mg*/*dL*)	150.0 [123.0–167.7]	122.2 [108.7–161.0]	0.184
*Hb1Ac* (*%*)	7.7 [7.0–8.7]	6.9 [5.9–7.8]	0.023
*Total Cholesterol* (*mg*/*dL*)	197.0 [157.5–217.0]	175.0 [134.0–193.0]	0.123
*HDL-c* (*mg*/*dL*)	48.5 [38.7–60.2]	43.0 [36.0–51.0]	0.102
*Triglycerides* (*mg*/*dL*)	129.5 [97.5–172.0]	162.0 [103.0–190.0]	0.310

## Data Availability

The data presented in this study are available on request from the corresponding authors.

## Supplementary Materials

The following supporting information can be downloaded at: https://www.mdpi.com/article/10.3390/antiox11101878/s1. Figure S1: (a) Serum soluble NOX2-derived peptide (sNOX2-dp), (b) serum H2O2, and (c) blood HBA (d) thromboxane B2 production, (e) soluble P-selectin levels and (f) soluble CD40 ligand before and 15 days after administration of GLP-1RA (*n* = 8) or Gliflozins (*n* = 8) in patients not taking acetylsalicylic acid (ASA) and all on statin therapy Data are expressed as mean ± SD. Intra- group significance: * *p* < 0.05.
