# Supplementary material for: The Sodium–Glucose Co-Transporter-2 (SGLT2) Inhibitors Reduce Platelet Activation and Thrombus Formation by Lowering NOX2-Related Oxidative Stress: A Pilot Study
Source: Antioxidants (Basel). 2022 Sep 22;11(10):1878. doi: 10.3390/antiox11101878 (PMC9598474; doi:10.3390/antiox11101878)
Supplement: Supplementary file 1 [file antioxidants-11-01878-s001.zip › antioxidants-1881963-supplementary.pdf]

Figure S1

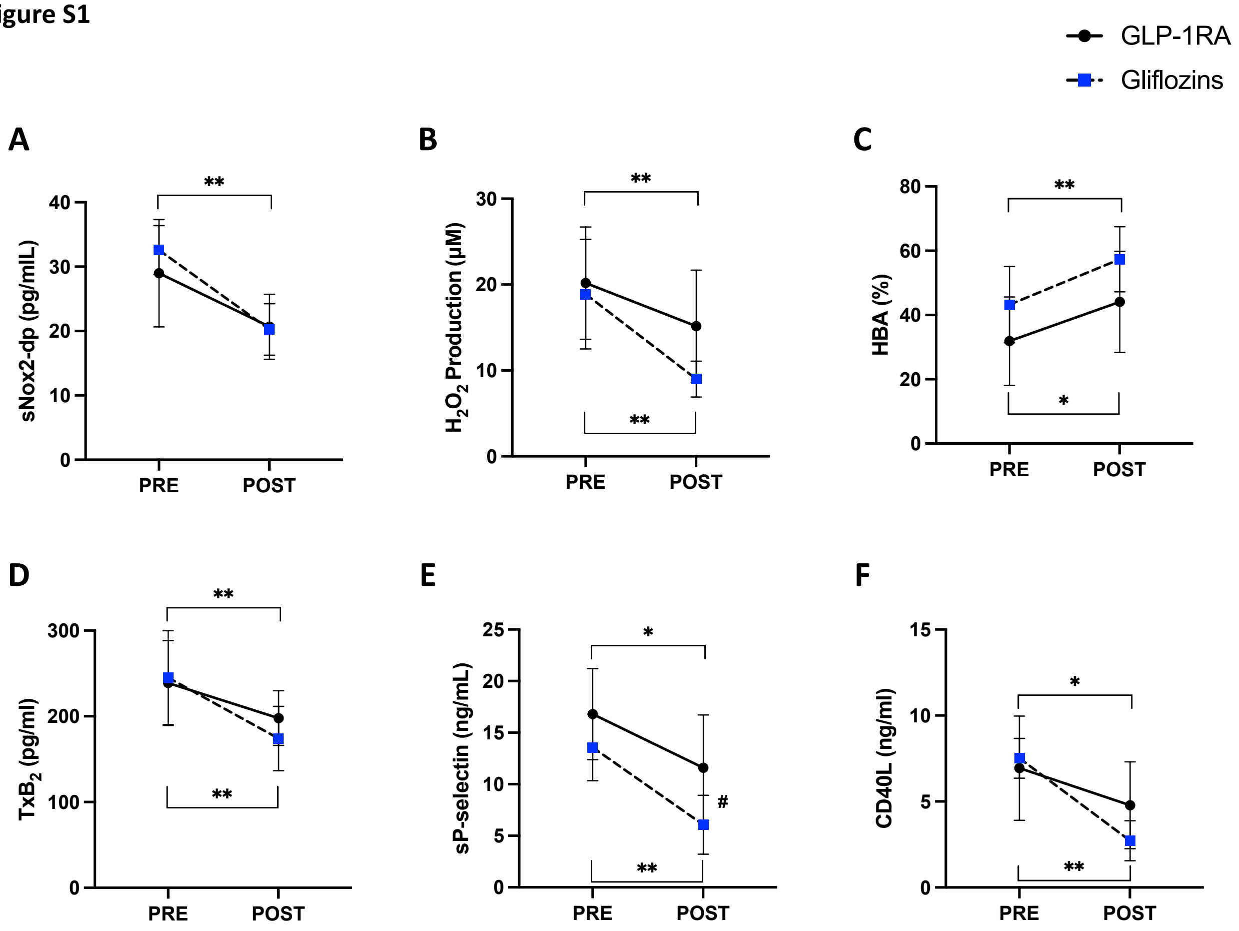

Figure S1. (a) Serum soluble NOX2-derived peptide (sNOX2-dp), (b) serum H_2O_2, and (c) blood HBA (d) thromboxane B_2 production, (e) soluble P-selectin levels and (f) soluble CD40 ligand before and 15 days after administration of GLP-1RA (n=8) or Gliflozins (n=8) in patients not taking acetylsalicylic acid (ASA) and all on statin therapy Data are expressed as mean±SD. Intra- group significance: *p<0.01; **p<0.001; inter-group significance: # p<0.05.
